# Linkage and Physical Mapping of Sex Region on LG23 of Nile Tilapia (*Oreochromis niloticus*)

**DOI:** 10.1534/g3.111.001545

**Published:** 2012-01-01

**Authors:** O. Eshel, A. Shirak, J. I. Weller, G. Hulata, M. Ron

**Affiliations:** * Robert H. Smith Faculty of Agriculture, Food and Environment, Hebrew University of Jerusalem, Rehovot 76100, Israel; † Institute of Animal Science, Agricultural Research Organization, Bet Dagan 50250, Israel

**Keywords:** sex region, linkage mapping, physical mapping, *Oreochromis niloticus*, microsatellite markers

## Abstract

Evidence supports that sex determination (SD) in tilapia is controlled by major genetic factors that may interact with minor genetic as well as environmental factors, thus implying that SD should be analyzed as a quantitative trait. Quantitative trait loci (QTL) for SD in *Oreochromis niloticus* were previously detected on linkage groups (LG) 1 and 23. Twenty-one short single repeats (SSR) of >12 TGs and one single nucleotide polymorphism were identified using the unpublished tilapia genome sequence on LG23. All markers showed two segregating alleles in a mapping family that was obtained by a cross between *O. niloticus* male (XY) and sex-reversed female (ΔXY) yielding 29 females (XX) and 61 males (XY and YY). Interval mapping analysis mapped the QTL peak between SSR markers ARO172 and ARO177 with a maximum F value of 78.7 (*P* < 7.6 × 10^−14^). Twelve adjacent markers found in this region were homozygous in females and either homozygous for the alternative allele or heterozygous in males. This segment was defined as the sex region (SR). The SR encompasses 1.5 Mbp on a single tilapia scaffold (no. 101) harboring 51 annotated genes. Among 10 candidate genes for SD that were tested for gene expression, anti-Müllerian hormone (*Amh*), which is located in the center of the SR, showed the highest overexpression in male *vs.* female embryos at 3 to 7 days postfertilization.

Sex determination (SD) can be controlled by one or more genetic factors, environment or their interactions, involved SD factors located on sex chromosomes and/or on either autosomes (Bull 1981). The sex chromosomes are characterized by both morphologically undifferentiated and differentiated homologs, in simple and multiple systems with male or female heterogamety. Studies on organisms with differentiated sex chromosomes, male heterogametic (mammals and fly) and female heterogametic (birds and reptiles), have shown interesting similarities between the two systems (XY/XX and ZW/ZZ) of sex determination (Ellegren 2011).

Teleost species are an interesting model for SD research, with a variety of SD systems and capability of producing viable hybrids between closely related species having different SD systems (Mank *et al.* 2006). Teleost fish have diverged from land vertebrates more than 450 million years ago, after they diverged from birds and mammals, but before they diverged from each other (Bellott *et al.* 2010). Moreover, these fish species experienced whole-genome duplication (ancestral tetraploidy), followed by variable-rate reduction of ploidy that significantly complicates the identification of orthologs (Kasahara *et al.* 2007). Bellott *et al.* (2010) compared zebrafish, Tetraodon, pufferfish, and medaka genomes to mammalian X or avian Z chromosome and reported that most orthologs to Z and X genes occupy separate portions of each fish genome.

Different aspects of tilapia SD have been explored because tilapias are an important aquaculture commodity (Devlin and Nagahama 2002). Their commercial production relies on all-male monosex culture, which so far has proved difficult to maintain in large-scale production facilities (Cnaani and Levavi-Sivan 2009). A better understanding of the genetic basis of SD in tilapia is needed to overcome these difficulties.

The differences in SD mechanisms among closely related tilapia species and the influence of the environment (Baroiller *et al.* 2009) suggest that SD should be analyzed as a quantitative trait using a markers-based QTL approach. Various sex-linked markers have been identified in *O. niloticus* and *O. aureus* (Lee *et al.* 2003, 2004; Shirak *et al.* 2002, 2006; Eshel *et al.* 2010) and mapped to different LG. In purebred *O. niloticus* and *O. niloticus* × *O. aureus* hybrids, the QTL were detected on LG1, LG23 (Lee *et al.* 2003; Eshel *et al.* 2010) and on LG3 (Lee *et al.* 2005), respectively. The SD QTL on LG23 was mapped within a confidence interval (CI) of 16–21 cM (Eshel *et al.* 2010; Figure 1B), which harbors the genes *Amh* and *Dmrta2* that are involved in the vertebrate SD cascade (Shirak *et al.* 2006, Figure 1A). The first assembly version of the unpublished tilapia genome, consisting of 5900 scaffolds, was recently released (Accession no. PRJNA59571). Using this information enabled us to refine the confidence interval of SD QTL on LG23 and find positional candidate genes for the sex master-key regulators in the Swansea stock of *O. niloticus*.

**Figure 1:**
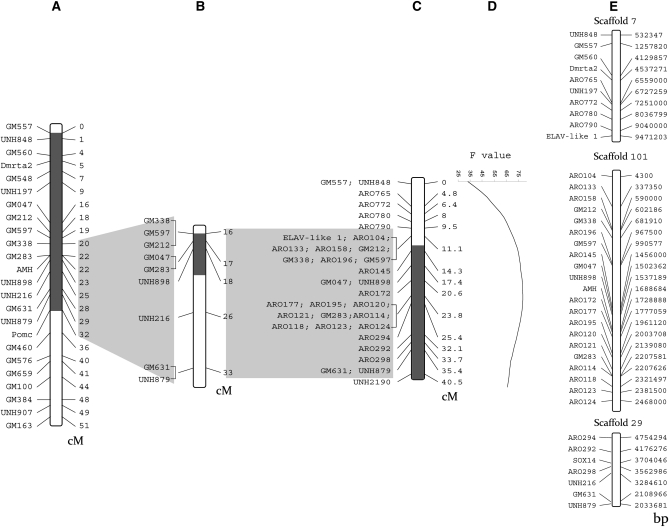
Linkage and physical map of SR on LG23 and interval mapping of the SD QTL. (A) LG23 genetic map by genotyping of *O. niloticus ♂* × *O. aureus* ♀ family (Shirak *et al.* 2006). (B) Sex determination QTL on LG23 for *O. niloticus* families based on nine SSR markers (Eshel *et al.* 2010). (C) Fine mapping of the QTL region with additional mapping family (sex reversed) with 33 markers. (D) Interval mapping of the QTL based on panel C. (E) LG23-related scaffolds. QTL, quantitative trait loci; SD, sex determination; SR, sex region; SSR, short single repeat.

The critical period for SD in tilapias is 0–18 days postfertilization (dpf). During this period, embryos are sensitive to androgens, estrogens, and precursors of steroids through immersion and dietary exposure (Devlin and Nagahama 2002). In a more recent study, Ijiri *et al.* (2008) demonstrated that differential expression of genes in XX and XY gonads of *O. niloticus* during the period of 9–10 dpf is critical for differentiation of primordial germ cells (PGC) into either ovary or testis. Rougeot *et al.* (2008) applied temperature treatment on all-female population embryos until hatching (3–4 dpf) and showed ∼20% phenotypic sex change of females to males. Furthermore, Palti *et al.* (2002) demonstrated by using genetic markers that sex-specific mortality occurs shortly after hatching. On the basis of these findings, we hypothesized that master regulation genes initiating the SD cascade should be expressed before the detectable differences in the PGCs.

Recent studies revealed that the major genes involved in the SD pathway are common to mammals and fish (Schartl 2004). Moreover, the upstream genes on the SD cascade may vary among organisms, but downstream components tend to be conserved (Charlesworth and Mank 2010). To study the onset of the SD cascade at early stages in embryonic development, we selected eight genes (*Lhx9*, *Amh*, *Foxl2*, Cyp*19a*, *Dmrt1*, *DAX1*, *Sox9a*, and *Sox9b*) with a known role in the SD pathway of other organisms (Birk *et al.* 2000; Shirak *et al.* 2006; Ijiri *et al.* 2008) and examined their expression from gastrula to late larval stage (2–9 dpf) in *O. niloticus* monosex progeny of XY males and XX females. Additionally, we analyzed two genes previously mapped to the SD region: *Sox14* (Cnaani *et al.* 2007) and *ELAVL1* (A. Shirak, unpublished data) with ambiguous similarity to known genes on the SD pathway. Gene expression profiles represent the primary level of integration between environmental factors and the genome, providing the basis for phenotypes, such as morphology and behavior. Therefore, we examined differences in candidate genes expression between genders during early embryonic development for initiation of the SD cascade in tilapia.

The objectives of this study were to refine the sex region on LG23 using both linkage and physical mapping and to identify candidate genes for SD in the region with differential expression at early embryonic development.

## Materials and Methods

Breeding of *O. niloticus* (Swansea stock) families used for this study was performed at the Agricultural Research Organization, Israel.

### Mapping family

The inheritance of gender in a cross between *O. niloticus* male (XY) and a sex-reversed neofemale (ΔXY) that yielded 29 females (XX) and 61 males (XY and YY) was validated by segregation of the sex-linked marker *UNH898* (Eshel *et al.* 2010).

### Monosex groups

To obtain all-female (XX) and all-male (XY) progenies, eggs of a single *O. niloticus* female (XX) were divided into two groups, and each group was artificially fertilized with either milt of a sex-reversed male (ΔXX) or milt of a genetically modified male (YY). Sex was determined at age of three months by gonadal squash of at least 100 individuals per each full-sib group (Mair *et al.* 1997).

#### Development of SSR markers:

We ran BLASTN search for the *Amh*, *Dmrta2*, and *Sox14* genes and for nine SSR markers that were previously mapped to LG23 (Lee *et al.* 2004; Shirak *et al.* 2006; Cnaani *et al.* 2008) against the unpublished tilapia genome (Accession no. PRJNA59571; http://cichlid.umd.edu/blast/blast.html). Hits were found in three scaffolds: no. 7, 101, and 29. We searched for tandem repeats of >12 TG in scaffold 7 (6,500,000–9,485,422 bp), in scaffold 29 (3,291,196–5,141,938 bp), and in the entire scaffold 101. We entered the sequence of 200 bp upstream and downstream of the TG repeats core to Primer3 software and developed 21 novel SSR markers. Polymorphism of these markers was tested in parents of the mapping family. To develop genetic markers in the vicinity of UNH216, we mapped in our family the marker UNH2190, which was derived from the Malawi cichlids hybrid *Metriaclima zebra* × *Labeotropheus fueleborni* and was mapped adjacent to UNH216 (Albertson *et al.* 2003).

#### Development of the SNP marker:

On the basis of partial cds sequence (GI: 93115149) of *O. mossambicus ELAVL1* (embryonic lethal, abnormal vision, Drosophila-like 1), we identified the SNP polymorphism A/G (Table 1) at nucleotide 391 in our mapping family.

**Table 1:** Primers for SSRs and *ELAV-like 1* gene used in this study and their locations on the unpublished tilapia genome scaffolds

Scaffold	Marker/Gene	Forward Primer	Reverse Primer
7	ARO765	CCTGAAACTCAGGCGCTGTA	GCTCTCACCAAGGTCAGCAA
	ARO772	GCCTTGTGCCACTGTAGGAG	AACCTGCCTCTCCTGGAATC
	ARO780	TGTGGGGTTTTTGAAGCCTA	GAAACCCCCTCTTCCTTGTG
	ARO790	TGAAGCAAACAGAGGCCATT	GCTGGGTGAGGGGTTTTGTA
	*ELAV-like 1*	GCTTTGATAAGAGGGCTGAGG	AGTTCCTGGCCTGGTTGG
		Extension primer: [AAAAAA]CAAACACCTGAACGGACACAC*^a^*	
29	ARO298	CAGACTGTCCCCATCCTCAA	AGGGAGCTGGATCTGCCTAA
	ARO292	TTGACTACCGGCTTGCATTC	GCCCGAACATAAGATGTCCA
	ARO294	TGCTCTCACTGCTGAGCAAA	CGCAAATGTTAGGCCAGAAA
101	ARO104	AAGACCCGTTCTTCGTCGTC	TTCATTCCACCTGCTCCAAA
	ARO133	GTGAGGCAAGTCCGGTTTCT	TGATCCACGGCGTATTGAGT
	ARO158	GTGGGCAAAAACAAGCCATT	TGTTTCAGTGTGAACGTGTGTG
	ARO196	GATTGTGGCCTGGTCAAGTG	TCCGTTTGTCTGCTGTGTGA
	ARO145	CAATGTGGCAATGTGTCCAA	CGGTGTCTCTGTGTCGTGTG
	ARO172	AGGCCTTTCATCGCTGTTTT	ACCCTGTAGATGAGCGCAAA
	ARO177	CCCTGCCCTGAACTACCTTC	GCTGCAAGCAAATGAAAAGC
	ARO195	CATGCTGATGGAGACCGATT	TCAAGACGCAATGGAGTGTG
	ARO120	AAGGGAAAGTGGCTCAGCTC	GTTGCTTCCCCACAGTTTCA
	ARO121	GGTGGGACTGTGGTGTATGG	GGTGGATTGCAAGCAACATT
	ARO114	AGGAGAAGTCGCAGGTGACA	GGCACAGTTGCCTGGTACAT
	ARO118	TGAATCTTCCCACAGCAACA	GTTGGTGCCAACAAAGCAAT
	ARO123	TTAATCCTGCCCACCTCTCC	AAGCAAAAGCATTTTCATGTTCA
	ARO124	CGAGCTGCTTTGTTGTCTGA	CGAACCGAAAATGAGAATGC

SSRs, short single repeats.

a [AAAAAA] is a stabilizing tail.

#### DNA extraction and genotyping of SSR and SNP markers:

DNA was isolated from fin samples by the “salting out” high-throughput procedure (Zilberman *et al.* 2006). The concentration of the DNA was quantified with NanoDrop spectrometer (NanoDrop Technologies, DE), and each DNA sample was diluted to a final concentration of ∼10 ng/µl. PCR amplification was performed in a total volume of 10 µl with Super-Therm Taq DNA polymerase (JMR Holding, London), mixture of 2 mM dNTPs of each nucleotide, and primer concentration of 10 pmol/µl (Metabion GmbH, Germany). PCR conditions were 3 min at 94°; 40 sec at 94°, 40 sec at 61.5°, 1 min at 72° for 30 cycles and 10 min at 72°. The mapping family was amplified for SSR markers, and genes with primers taken from NCBI database or designed based on scaffold sequence (detailed list in Table 1), where one primer in each pair was 5′ end-labeled by HEX, TET, or FAM fluorescent dyes (Operon Technologies, Alameda, CA). Size calling of PCR products was determined using ABI GeneMapper software version 4.0 (Applied Biosystems, Foster City, CA) after electrophoresis in a capillary gel on ABI-3130 apparatus. Sequencing and SNaPshot reaction for genotyping of SNP markers were also carried out on ABI-3130 according to the manufacture instructions using the primers specified in Table 1. (For additional data, see supporting information, File S1.)

#### Linkage and interval mapping:

The linkage map for LG23 using segregating markers in our mapping family was reconstructed by CRIMAP software (http://linkage.rockefeller.edu/soft/crimap/).

The interval mapping was based on a nonlinear regression using the method of Knott *et al.* (1996), with the program developed by Spelman *et al.* (1996). The test statistic and locus effects were evaluated at 1 cM intervals. The 95% confidence intervals (CI) for the QTL location and effect were determined by generation of 200 bootstrap samples.

#### Identification of genes and annotation:

Annotation of genes positioned in the SR was performed by combining three bioinformatics resources: (1) EST contigs assembled via MIRA program with BLASTN (Lee *et al.* 2010); (2) comparative mapping to other fish genomes by BLASTX; and (3) BouillaBase annotation using Maker Gene pipeline (http://cichlid.umd.edu/cgi-in/gb2/gbrowse/Tilapia_broad_scaffolds_v1/?source=Tilapia_broad_scaffolds_v1).

#### Comparative mapping:

After determining the boundaries of the SR in LG23, we detected 39 annotated genes in the unpublished tilapia genome database (http://cichlid.umd.edu/cgi-bin/gb2/gbrowse/Tilapia_broad_scaffolds_v1/?source=Tilapia_broad_scaffolds_v1) using Maker pipeline. Further analysis between tilapia and stickleback group VIII (15.4–16.7 Mbp) (http://www.ensembl.org/index.html) identified all 39 genes in the same order in both genomes. On the basis of this high level of orthology, we used stickleback orthologous region on UCSC genome browser (http://genome.ucsc.edu/) as anchor for similarity with Tetraodon (chr1:13.4–14.7 Mbp), medaka (chr4:7.4–8.8 Mbp), fugu (scaffold 25:0.4–1 Mbp), zebrafish (chr22:19.1–21.6 Mbp), and human (chr19p13.3: 1.5–5 Mbp), and we detected 12 additional genes in tilapia.

#### RNA extraction and qPCR:

A pool of 20–30 embryos from each gender were placed in RNAlater reagent (Qiagen) to stabilize the RNA and then stored at −20° until RNA extraction. Total RNA was extracted (miRNeasy Mini kit, QIAGEN) and analyzed with Agilent 2100 Bioanalyzer (Agilent Technologies, Palo Alto, CA). Synthesis of cDNA was done with SuperScript II (Invitrogen) according to the manufacturer’s instructions. Three biological and three technical repeats of qPCR were performed using Absolute Blue SYBR Green Rox mix (Thermo Scientific, WI). The primers were designed so at least one strand was specific to an intron-exon boundary (Table 2). The efficiency of the PCR reaction (linear equation: y = slope + intercept) was measured in triplicate on serial dilutions of the same cDNA sample (pool of RT-RNA samples). Efficiencies (E) of qPCR for each reaction were calculated using the following equation: E = [10^(1/slope)^]^-1^. Melting-curve analysis was performed for each gene for specificity of qPCR products. The relative amount of the target RNA, called the input amount (IA) according to User Bulletin #2 ABI (PRISM7700 Sequence Detection System, Applied Biosystems), was determined by comparison with the corresponding standard curve for each sample. The IA values were calculated as follows: IA = [10^((Ct - intercept)/slope)^], where Ct is the cycle threshold for unknown sample. Each transcript level was normalized by division with the expression values of the *GAPDH* gene, which was used as an internal standard. Final results were analyzed by Student *t*-test. (For additional data, see File S2.)

**Table 2:** Primers for genes analyzed by qPCR

Gene	Forward Primer	Reverse Primer
*Amh*	GCACCCAGCTGCAGTACAC	GTGGGAGGTCAAAGGTCAAC
*Cyp19a1a**^a^*	GCATAGGCACAGCCAGCAAC	GTGCACTGCTGAAGATCTGCTTAGTA
*Dax1*	CAGATCTGGAGGGTTTGC	GATGGATCAGCCTGACGTG
*Dmrt1**^a^*	CGGCCCAGGTTGCTCTGAG	CCAACTTCATTCTTGACCATCA
*ELAV-like 1*	CAGGCTTCAGGTCTGTCACG	GTGTCCGTTCAGGTGTTTGA
*Foxl2*	CACGACCAAGGAGAAAGAGC	TGGCAATGAGAGCGACATAG
*Lhx9*	GATTACTACAGGTTCTCCGTGCAG	TCAGGTGATACACGGAGTCG
*Sox14*	TGCTCAAGAAGGACCGTTACG	AAGAGCCCAAAAGAGAGTCCG
*Sox9a*	GCAAACTTTGGAGATTGCTCA	TCGGGGTGATCCTTCTTATG
*Sox9b*	GAGAGCATTCAGGTCAGTCACA	TCAGATCAGCTTTGCTGGAG
*GAPDH*	GGCATCGTGGAAGGTCTCAT	CATTTTACCAGAGGGCCCGT

a Ijiri *et al.* (2008).

#### Gene expression in SD-associated organs:

To retrieve available gene expression data for all genes embedded within the SR on LG23 we used the “Gene Atlas” expression data for mammals at BioGPS (Su *et al.* 2004; Wu *et al.* 2009; http://biogps.org/#goto=welcome). Differential expression was determined for individual genes in organs relevant for the SD pathways in tilapia such as brain, testis and pituitary.

## Results

### Mapping new markers on LG23

Two alleles were found for each of the novel 21 SSR markers in the parents of the mapping family. These markers were designated as ARO markers (Table 1) that were physically mapped to scaffolds 7, 101, and 29 and linkage mapped to an interval of 30 cM of LG23 (Figure 1C).

### Linkage and physical mapping of the QTL on LG23

**Figure 2:**
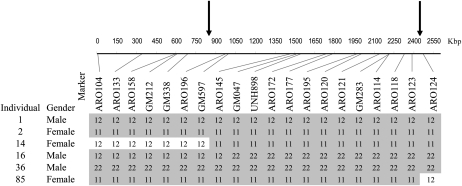
Determination of boundaries of the SR on scaffold 101 based on genotyping data of SSRs for selected individuals. Heterozygous genotypes of females contributed to the reduction of the SR interval delimited by arrows. The two homozygous genotypes are denoted “11” and “22”; the heterozygous is denoted as “12.” Females have the 11 genotype for all markers within the SR, whereas males have either 12 or 22. The genotypes’ segments corresponding to gender for each individual are denoted with shading. SR, sex region; SSRs, short single repeats.

In Figure 1, the QTL interval mapping for SD on LG23 is presented based on the reference mapping family (Figure 1A) and *O. niloticus* families (Figure 1B). In the current study, 33 genetic markers were analyzed, including the new SSR markers added (Figure 1C). Interval mapping analysis mapped the SD QTL region to 13–40 cM with a maximum F value of 78.7 (*P* < 7.6 × 10^−14^) at 22 cM (Figure 1D). This region was localized to scaffolds 7, 101, and 29. Physical mapping of the scaffolds with the newly developed markers narrowed down the SR to scaffold 101 between markers GM597 and ARO124 from 990,577 to 2,468,000 bp (Figures 1E and 2). The *Amh* gene is located between these markers. The scaffolds relating to LG23 and the physical map of markers are given in Figure 1E based on the unpublished tilapia genome sequence.

The SR on scaffold 101 was inferred from genotypes for SSRs of selected individuals (Figure 2); 12 adjacent markers found in this region were homozygous in females and either homozygous for the alternative allele or heterozygous in males. This segment was defined as sex region. Markers flanking the SR were heterozygous in females, thus reducing the SR interval to 1.5 Mbp between GM597 and ARO124. The boundaries of the SR are marked by arrows.

### Gene expression at early developmental stages

**Figure 3:**
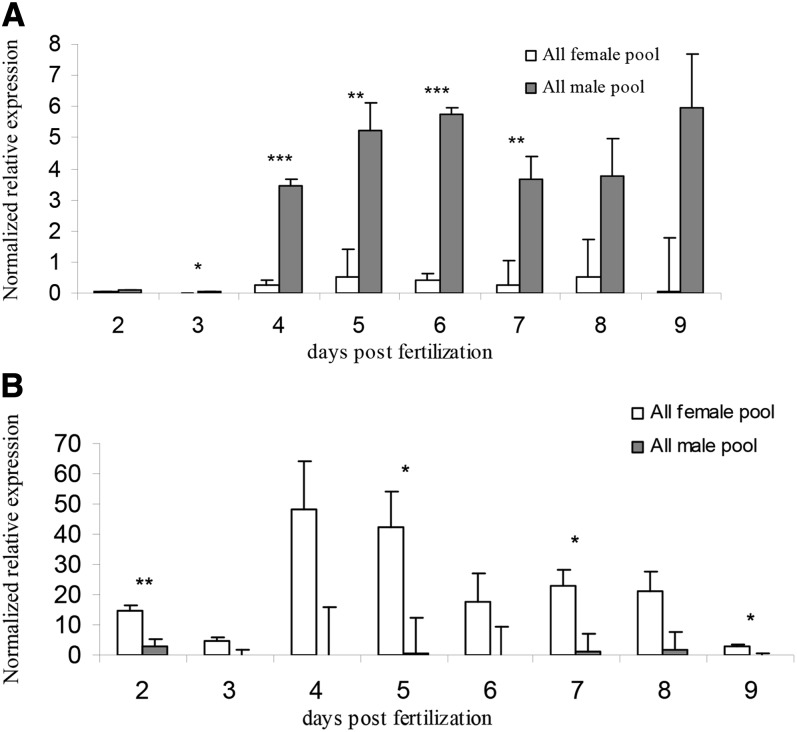
Normalized relative expression of *Amh* (A) and *ELAVL1* (B) for all-male (gray) and all-female (white) pools at 2–9 dpf. Deviation bars represent standard errors and asterisks represent levels of significance for sex-specific expression differences: ^*^P ≤ 0.05, ^**^P ≤ 0.01, and ^***^P ≤ 0.001. dpf, days postfertilization. A is presented in the lower part of Figure 3.

Expression analysis of 10 SD-related genes and two genes mapped within the SD QTL on LG23 was performed for embryos of known type (XX or XY) during 2–9 dpf. No significant differences between genders were found for cyp*19a*, *Dmrt1*, or *Dax1*. Significant sex-differential expression was detected for the remaining 7 genes as presented in Table 3. Figure 3A presents the continued elevation in significance for gender-specific differences for *Amh* expression from 3 dpf (*P* = 0.03) to 7 dpf (*P* ≤ 0.01). The Y axis indicates normalized expression values, whereas each bar along the X axis indicates a sample. This gene showed the highest sex-differential expression among all 10 tested genes. Significant sex difference was found for *Lhx9* expression (*P* = 0.0002) at 2 dpf, equivalent to the developmental stage of segmentation (Fujimura and Okada 2007) but not later in the embryonic development. Likewise, sex-differential expression was detected for *ELAVL1* (*P* < 0.01) at age of 2 dpf but was attenuated at 5, 7, and 9 dpf (*P* ≤ 0.05) (Figure 3B). Gender-specific expression differences for the other 4 genes (*Foxl2*, *Sox9a*, *Sox9b*, and *Sox14*) were detected at later developmental stages (6–9 pdf).

**Table 3:** Level of normalized relative expression ± SD and statistical significance of sex-specific differences for gene candidates for SD in embryos at 2 to 9 dpf

Gene	dpf	All Female	All Male	Probability
*ELAVL1*	2	14.5 ± 2.2	3.2 ± 2.1	**
	5	42.1 ± 11.8	0.5 ± 11.8	*
	7	22.8 ± 5.8	1.2 ± 5.4	*
	9	2.7 ± 0.6	0.2 ± 0.6	*
*Amh*	3	0.02 ± 0.005	0.04 ± 0.005	*
	4	0.2 ± 0.2	3.5 ± 0.2	***
	5	0.5 ± 0.9	5.2 ± 0.9	**
	6	0.4 ± 0.2	5.8 ± 0.2	***
	7	0.3 ± 0.7	3.7 ± 0.7	**
*Lhx9*	2	3.1 ± 0.18	0.8 ± 0.2	***
*Sox9a*	7	1.6 ± 0.17	0.9 ± 0.2	*
*Sox9b*	7	6.3 ± 0.6	4 ± 0.6	*
*Foxl2*	8	0.9 ± 0.06	0.6 ± 0.06	*
*Sox14*	9	0.3 ± 0.07	0.55 ± 0.06	*

Asterisks represent levels of significance for sex-specific expression differences: ^*^P ≤ 0.05, ^**^P ≤ 0.01, and ^***^P ≤ 0.001. dpf, days post fertilization.

### Characterization of genes positioned within the SR

Fifty-one genes were identified within the SR and are presented in Table 4. Thirty-nine genes had expression data in a variety of 91 tissues of mammals in the BioGPS database. We focused on three SD-related organs that are relevant in the tilapia SD cascade: brain, testis, and pituitary. Interestingly, 17 out of the 39 genes showed overexpression in the brain; expression of 15 of these genes exceeded the median expression by over 3-fold. Thirteen genes were found relevant to SD following a literature survey. After removing 3 genes with no expression data, 4 out of the remaining 10 genes showed overexpression in at least one SD-associated organ (*Notch2*, *PIAS4*, *ZBTB7*, or *CELF5*).

**Table 4:** SD-related data for annotated genes in the SR on scaffold 101 between 990,577 and 2,468,000 bp

			Scaffold 101 (bp)			Gene Expression		
	Gene or Symbol	Accession or Ensembl No.	Start	End	SD-related Publications	Brain	Testis	Pituitary
1	*PLIN3*	NP_001167399.1	1187253	1192169				
2	*ZFAND6*	XP_002199446.1	1194645	1198048			√√	
3	*FAM108C1*	XP_001342996.2	1205385	1210274				
4	*RGL1*	NP_991200.1	1216521	1224747		√√	√	
5	*GLT25D2*	ENSGACT00000016402	1245440	1265427		√√		
6	Novel protein	ENSGACT00000016408	1273512	1277395		ND		
7	*C1orf21*	ENSGACT00000016410	1273570	1287700		√√		
8	*EDEM3*	XP_688275.4	1305007	1311942				
9	*NPL*	NP_001133311.1	1314238	1318468				
10	*SEC22B*	ACM09163.1	1328257	1332317				√√
11	*NOTCH2**^a^*	NP_001108566.1	1348777	1380740	Zhu *et al.* 2007		√√	
12	*SLC35A3*	ACN10890.1	1384970	1391950				
13	*FAM78B*	CAQ14615.1	1390770	1404535		ND		
14	*C19orf60*	NP_001158740.1	1406814	1410851		√√		
15	*CRLF1*	NP_001002650.1	1412045	1421020				
16	*TMEM59L*	ENSGACT00000016558	1422768	1431927		√√		
17	Novel protein	ENSGACT00000016563	1431927	1433134		ND		
18	*SSBP4*	NP_001018403.1	1502998	1511875		√√		
19	*FKBP8*	NP_001133417.1	1569993	1579098		√√		
20	*ELL**^a^*	NP_956001.1	1603737	1613759	Zhou *et al.* 2009			
21	*DOT1*	CAP09616.1	1667805	1679590				
22	*Amh*	ABS58513.1	1688658	1695299			√√	
23	Novel protein	ENSGACT00000016737	1691664	1695317		ND		
24	*OAZ1*	NP_001134904.2	1696653	1701716		√√		
25	*dkey-3k20.4*	ENSGACT00000016747	1705254	1704891		ND		
26	*ORG**^b^*	NP_001093540.1	1709490	1716883	Dai *et al.* 2009	ND		
27	*LINGO3*	ENSGACT00000016753	1769930	1771737				
28	Novel protein	ENSGACT00000016755	1783055	1787782		ND		
29	*ATP8B3*	XP_003201102.1	1808332	1824572				
30	*ONECUT3*	ENSGACT00000016776	1832236	1848746		ND		
31	*PIAS4**^a^*	AAH57528.1	1876824	1885646	Hsieh *et al.* 2009	√√	√	
32	*MAP2K2**^a^*	XP_002761634.1	1892758	1902715	Murakami *et al.* 2001			
33	*ZBTB7**^a^*	CAK04316.1	1917084	1923570	Gailey *et al.* 2006	√√		
34	*TCF3**^a^*	NP_001187227	1948472	1961119	Zhu *et al.* 2007			
35	*QCR10**^a^*	ACQ58208.1	1968852	1973040		ND		
36	*MBD3b*	CAK10918.1	1973187	1979287	Kaji *et al.* 2006	ND		
37	*MYO5b*	CAK10917.1	1980557	1993470				
38	*UNC13A*	NP_001038630.1	1997106	2036658		√√		√
39	*HMG20b**^a^*	NP_001018387.1	2039148	2044881	Sumoy *et al.* 2000			
40	*EEF2*	NP_956752.2	2047515	2063389				
41	*SNORD37*	ENSGACT00000029482	2050851	2051406		ND		
42	*Rxfp3**^b^*	NP_001077348.1	2080930	2081856	Wilson *et al.* 2009			
43	*CREB3*	NP_001018509.1	2088906	2099759		√√		√
44	*CELF5**^a^*	NP_001124260.1	2263338	2289589	Ladd *et al.* 2001	√√		
45	*RGMA*	NP_001133864.1	2299255	2306831		√√	√√	
46	*HSD11B1L**^b^*	NP_001098261.1	2342628	2365654	Ozaki *et al.* 2006	ND		
47	*QIL1*	ACI69344.1	2365927	2368604		√	√	
48	*SPIN1*	XP_001339043.4	2370662	2372447		√		
49	*CFD*	ACI69308.1	2392555	2404506			√	
50	*BTBD8**^a^*	EAW73104.1	2406880	2407219	Couderc *et al.* 2002			
51	*CYLIP1*	ACN58730.1	2459356	2488052		√√		

√ indicates 2–3× from median expression in BioGPS; √√ indicates >3× from median expression in BioGPS; ND, no data; SD, sex determination; SR, sex region.

a Gene related to SD in transcriptional processes.

b Gene associated with gonad development/function.

### Comparative mapping

Comparative analysis of the genes positioned within the SR detected high level of orthology between tilapia and six different species. Within <1.3 Mbp region of stickleback, Tetraodon, fugo, zebrafish, medaka, and human, 40, 39, 29, 29, 29, and 21 orthologous genes, respectively, have been found in the same order. GO term enrichment analysis of these genes with DAVID software (Huang *et al.* 2009) yielded 4 genes, *Notch2*, *ELL*, *Amh*, and *TCF*, which are involved in biological processes of cell differentiation, cellular, and anatomical structure development based on zebrafish background of 8389 genes.

## Discussion

Different SD systems with remarkable variation have been observed in teleosts (Volff and Schartl 2002). Evidence supports that sex determination (SD) in tilapia is controlled by major genetic factors that may interact with minor genetic as well as environmental factors, thus implying that SD should be analyzed as a quantitative trait.

QTL for SD in *Oreochromis niloticus* were previously detected on LG1 and LG23 (Lee *et al.* 2003; Cnaani *et al.* 2004; Eshel *et al.* 2010). In the present study, interval mapping analysis using 33 markers on LG23 detected the QTL peak between two adjacent genetic markers: ARO172 and ARO177. However, the confidence interval was still rather large between 5 cM (Eshel *et al.* 2010; 156 individuals) and 30 cM in the current study. Thus, mapping QTL to confidence interval < 5 cM is not a viable option using genetic markers and segregating families of moderate size (Ron and Weller 2007). However, using physical mapping based on the unpublished tilapia genome sequence, all 26 markers in the QTL were physically mapped to three scaffolds on LG23. Furthermore, recombinations in two females were used to identify the boundaries of the SR between markers GM597 and ARO124 on a single scaffold (no. 101; Figure 2). This explains the lack of power of the interval mapping that is based on bootstrap analysis of a family of 90 individuals of which only two are informative. Figure 2 demonstrates the distinct contrast of genotypes for markers between genders in the specific sex region. The absence of recombination along a region of 12 genetic markers may reflect the moderate size of the family, but it also conforms to the theory that the evolution of sex chromosomes involves suppressed recombination between homologous chromosomes to maintain sex-related gene blocks (Bergero and Charlesworth 2009). The SR encompasses 1.5 Mbp harboring 51 annotated genes. Our assumption that the SR harbors sex-related or male-determining genes is strengthened by the conservation of this region in other teleost fish. Out of 51 genes that were positioned within the SR, 40 and 39 orthologous genes have been found within <1.3 Mbp region of stickleback and Tetraodon, respectively. Information from the literature indicates the putative role of 13 out of the 51 genes in SD: 10 genes in transcriptional processes related to SD and 3 in gonadal development and function (Table 4).

We examined expression of genes in the SD pathways at early developmental stages of tilapia. Previous studies on SD-related gene expression in tilapia focused on brain, PGS, and gonads (Ijiri *et al.* 2008; Poonlaphdecha *et al.* 2011). The results from our study on expression data of 10 candidate genes indicate that the onset of the SD cascade begins at 2 dpf at the gastrulation stage, based on overexpression of *Lhx9* and *ELAVL1* in females. *Lhx9* was found to be essential for mouse gonad formation (Birk *et al.* 2000). *ELAVL1* is a member of CELF proteins implicated in cell-specific and developmentally regulated alternative splicing (Ladd *et al.* 2001). Additional SD-related genes were *Sox9*, which is necessary and sufficient to cause testicular differentiation in mammals (Vidal *et al.* 2001). Likewise, *Foxl2* plays a role in ovarian sex differentiation and has been suggested to function as a repressor of the male pathway during ovarian development prophase (Ottolenghi *et al.* 2005). Significant differences in expression between genders for *Sox9a*, *Sox9b*, *Foxl2*, and *Sox14* genes were detected in later stages of embryonic development and may indicate their downstream role in the SD cascade. We detected higher expression of *SOX9* in females than in males at 7 dpf, in contrast to the results of Ijiri *et al.* (2008) of higher expression in male gonads at 37–70 days posthatching. *Foxl2* was also highly expressed in females at 8 dpf as was previously reported (Ijiri *et al.* 2008). Among 10 ten candidate genes, *Amh*, which is located in the center of the SR, showed the highest expression in male *vs.* female embryos. Our observation was supported by Poonlaphdecha *et al.* (2011) who reported on dimorphic expression of *Amh* between genders in adult gonads and brains as well as in embryo heads at 10 and 15 dpf. GO term enrichment analysis detected 4 genes, including *Amh*, that are involved in biological processes of cell differentiation, cellular development, and anatomical structure development. Genes playing a role in SD initiation with dimorphic expression between genders may be considered as candidate genes and should be further investigated.

To test the role of *Amh* and other candidate genes in SD of tilapia, targeted strategies could be considered, such as (i) mutant detection in candidate genes, as performed in zebrafish (Demarest *et al.* 2011); (ii) gene silencing using siRNA technology, as applied in the giant freshwater prawn (Ventura *et al.* 2009); and (iii) transgenesis using the *Tol2* system, which was demonstrated for Nile tilapia (Fujimura and Kocher 2011). Large-scale experiments might involve (i) genomic mutagenesis together with sex reversal, phenotypic mutant screening, and sequence analysis, as was applied in a medaka SD study (Otake *et al.* 2006); and (ii) a whole-transcriptome scan for gene expression at early embryonic development to identify the key regulators of SD. A complete computational approach was pursued to design a 44k features microarray (O. Eshel, unpublished data) based on the unpublished tilapia genome sequence annotation and EST libraries (Lee *et al.* 2010) for construction of the full tilapia gene list.

## Supplementary Material

Supporting Information

Corrigendum
