# Supplementary material for: Linkage and Physical Mapping of Sex Region on LG23 of Nile Tilapia (Oreochromis niloticus)
Source: G3 (Bethesda). 2012 Jan 1;2(1):35–42. doi: 10.1534/g3.111.001545 (PMC3276181; doi:10.1534/g3.111.001545)
Supplement: Supporting Information [file supp_2_1_35__index.html]

Supporting Information 

# Linkage and Physical Mapping of Sex Region on LG23 of Nile Tilapia (*Oreochromis niloticus*)

## Supporting Information for Eshel *et al.*, 2012

**Files in this Data Supplement:**

- File S1 - Supporting data (.xls, 64 KB)
- File S2 - Supporting Data (.xls, 252 KB)
