# Supplementary material for: Linkage and Physical Mapping of Sex Region on LG23 of Nile Tilapia (Oreochromis niloticus)
Source: G3 (Bethesda). 2012 Jan 1;2(1):35–42. doi: 10.1534/g3.111.001545 (PMC3276181; doi:10.1534/g3.111.001545)
Supplement: Corrigendum [file supp_2_1_35_v2_index.html]

Corrigendum 

# Linkage and Physical Mapping of Sex Region on LG23 of Nile Tilapia (*Oreochromis niloticus*)

## Corrigendum for Eshel *et al*., *G3* 2 (1) 35-42

**Files in this Data Supplement:**

- Corrigendum - Corrigendum for Eshel *et al*., *G3* 2 (1) 35-42.
