# Supplementary material for: Linkage and Physical Mapping of Sex Region on LG23 of Nile Tilapia (Oreochromis niloticus)
Source: G3 (Bethesda). 2012 Jan 1;2(1):35–42. doi: 10.1534/g3.111.001545 (PMC3276181; doi:10.1534/g3.111.001545)
Supplement: Corrigendum [file supp_2.1.35_Eshel_corrigendum_FINAL.pdf]

Corrigendum for Eshel *et al.*, *G3* 2 (1) 35-42.

G3: *Genes* **|** *Genomes* **|** *Genetics*, Vol 2, 35-42, January 2012, Copyright © Eshel *et al*.

CORRIGENDUM

In the article by O. Eshel, A. Shirak, J. I. Weller, G. Hulata, and M. Ron (*G3* 2: 35-42) entitled “Linkage and Physical Mapping of Sex Region on LG23 of Nile Tilapia (*Oreochromis niloticus*),” the statement “A is presented in the lower part of Figure 3” has been added to the Figure 3 legend for clarification.
